# How Do Tsetse Recognise Their Hosts? The Role of Shape in the Responses of Tsetse (*Glossina fuscipes* and *G. palpalis*) to Artificial Hosts

**DOI:** 10.1371/journal.pntd.0001226

**Published:** 2011-08-02

**Authors:** Inaki Tirados, Johan Esterhuizen, Jean Baptiste Rayaisse, Abdoulaye Diarrassouba, Dramane Kaba, Serge Mpiana, Glyn A. Vale, Philippe Solano, Michael J. Lehane, Stephen J. Torr

**Affiliations:** 1 Natural Resource Institute, University of Greenwich, Chatham, Kent, United Kingdom; 2 Vector Group, Liverpool School of Tropical Medicine, Liverpool, United Kingdom; 3 Centre International de Recherche et Développement sur l'Élevage en Zone Subhumide, Bobo-Dioulasso, Burkina Faso; 4 Institut Pierre Richet, Abidjan, Côte d'Ivoire; 5 Laboratoire Vétérinaire Central de Kinshasa, Kinshasa, Democratic Republic of Congo; 6 Institut de Recherche pour le Développement, UMR 177 IRD-CIRAD, Montpellier, France; National Institute of Allergy and Infectious Diseases, United States of America

## Abstract

Palpalis-group tsetse, particularly the subspecies of *Glossina palpalis* and *G. fuscipes,* are the most important transmitters of human African trypanomiasis (HAT), transmitting >95% of cases. Traps and insecticide-treated targets are used to control tsetse but more cost-effective baits might be developed through a better understanding of the fly's host-seeking behaviour. Electrocuting grids were used to assess the numbers of *G. palpalis palpalis* and *G. fuscipes quanzensis* attracted to and landing on square or oblong targets of black cloth varying in size from 0.01 m^2^ to 1.0 m^2^. For both species, increasing the size of a square target from 0.01 m^2^ (dimensions = 0.1×0.1 m) to 1.0 m^2^ (1.0×1.0 m) increased the catch ∼4x however the numbers of tsetse killed per unit area of target declined with target size suggesting that the most cost efficient targets are not the largest. For *G. f. quanzensis,* horizontal oblongs, (1 m wide×0.5 m high) caught ∼1.8x more tsetse than vertical ones (0.5 m wide×1.0 m high) but the opposite applied for *G. p. palpalis*. Shape preference was consistent over the range of target sizes. For *G. p. palpalis* square targets caught as many tsetse as the oblong; while the evidence is less strong the same appears to apply to *G. f. quanzensis.* The results suggest that targets used to control *G. p. palpalis* and *G. f. quanzensis* should be square, and that the most cost-effective designs, as judged by the numbers of tsetse caught per area of target, are likely to be in the region of 0.25×0.25 m^2^. The preference of *G. p. palpalis* for vertical oblongs is unique amongst tsetse species, and it is suggested that this response might be related to its anthropophagic behaviour and hence importance as a vector of HAT.

## Author Summary

While the numbers of cases of human African trypanosomiasis (HAT) is now less than 10,000 reported cases per year, progress against the tsetse species that spread the disease is poor, with ∼10 million square kilometres of sub-Saharan Africa still being infested. This widespread persistence of vectors and reservoir hosts threatens the long-term sustainability of recent gains against HAT. Better progress against the vector would be achieved by developing cheap, effective and practical methods of tsetse control. Toward this end, we are improving the design of insecticide-treated targets to attract and kill tsetse. Here we show that for two important vectors of HAT, *Glossina palpalis palpalis* in Côte d'Ivoire and *Glossina fuscipes quanzensis* in the Democratic Republic of Congo, small (between 0.25 m and 0.5 m square) targets of black cloth with equally sized panel of fine black netting are ∼10x more cost-effective than the larger (∼1 m square) targets or traps commonly in use.

## Introduction

Between 1997 and 2006, about 250,000 new cases of Human African Trypanosomiasis (HAT, or sleeping sickness) were reported [1]. For >95%% of these cases, the disease started with a bite from one of four subspecies of tsetse: *Glossina palpalis gambiensis* (in Guinea and Côte d'Ivoire), *G. p. palpalis* (in Benin, Nigeria, western Cameroon, Equatorial Guinea, Gabon, south-western Republic of Congo, south-western Democratic Republic of Congo and western Angola), *G. fuscipes fuscipes* (in eastern Cameroon, Central African Republic, western Republic of Congo, northern DRC, Sudan, Uganda), and *G. f. quanzensis* (in southern DRC and northern Angola) [2].

Efforts to tackle HAT have been based largely on case-detection and treatment in humans [1] rather than vector control, largely because methods for controlling tsetse are too expensive and logistically demanding [3]. The use of natural (insecticide treated cattle) or artificial (traps and insecticide-treated targets, sometimes baited with attractants) baits are the only techniques that might be applied by local communities [3]–[7]. However, their wider use is constrained by the low densities of livestock in HAT-affected areas [8] and/or the poor performance of artificial baits for Palpalis-group tsetse. In contrast to Morsitans-group tsetse, Palpalis-group species are less responsive to host odours [9] and hence artificial baits must be deployed at densities that are not affordable or sustainable for poor people. However, recent results have revived the prospects for the use of cost-effective baits against HAT.

The performance of artificial baits can be enhanced by the use of attractants which double the capture rates [10], [11]. Second, several studies [12]–[14] suggest that significant improvements in cost-effectiveness of baits for vectors of HAT might be achieved through the exploitation of the visual responses to hosts. For instance, studies of *G. f. fuscipes* in Kenya showed that reducing the size of the target from 1 m^2^ to 0.125 m^2^ only halved the number of tsetse that contacted the target thereby giving a four-fold improvement in the tsetse killed per dollar spent on cloth [12]. Of course, the material cost of targets is only part of the total cost of deploying them and we would expect that the logistical costs of deploying targets will also be considerably reduced when using tiny targets.

The relationship between a target's size and the number of Palpalis-group tsetse differs markedly from that of Morsitans-tsetse – for the latter smaller targets are not cost-effective [15]. This suggests that there might be other differences in the visual responses of Palpalis- and Morsitans-group which tsetse which might be used to develop better targets.

Hitherto, research to improve target design has focussed on responses to colour [16]–[20] and size [12] but not shape. However, studies of Morsitans-group tsetse suggest that shape is important. Various studies in Zimbabwe have shown that more *G. morsitans* and *G. pallidipes* are attracted to and land on horizontal-oblongs rather than vertical ones [21], [22]. This shape recognition is thought to enable tsetse to discriminate their hosts from the environment. Important hosts, such as warthog and buffalo, are horizontal oblongs living in a visual environment of vertical oblongs formed by savannah woodland. This attraction to horizontal shapes is also thought to explain, at least in part, why Morsitans-group tsetse are not attracted to humans [21].

Intriguingly, Palpalis-group tsetse have a wider range of hosts which includes humans [23]–[25] and they are not confined to savannah woodlands. Hence, these species might be expected to display different behavioural responses to shape. An understanding of these responses would contribute to the rational development of more cost-effective designs of target. Consequently, this study assessed the responses of *G. p. palpalis* and *G. f. quanzensis* to targets of various shape. Separate studies have shown that target size has important effects on the numbers of tsetse attracted to and landing on a target [12]–[14]. We therefore also assessed whether responses to shape were affected by target size.

## Materials and Methods

### Study sites

#### *G. p. palpalis*

Studies were carried out in Côte d'Ivoire between December 2009 and February 2010 (the dry season) at sites near Azaguié (05°40′N, 04°02′W), ∼45 km north of Abidjan. Scattered patches of the original rain forest are interspersed with farms growing various crops including banana, coffee, cocoa, rubber and oil palm. Potential hosts for tsetse in the area include monitor lizard (*Varanus niloticus*), dwarf crocodile (*Osteolaemus tetraspis*), domestic pigs, cattle and humans [11].

#### *G. f. quanzensis*

Field studies of *G. fuscipes quanzensis* were undertaken in the Democratic Republic of the Congo (DRC) in the valley of the river Lukaya, ∼30 km south of Kinshasa (04°29′S, 15°18′E). Experiments were conducted during the dry season, between July and August in 2009 and 2010. Experimental sites were located in fields, where cassava and other subsistence crops were cultivated. Livestock, particularly pigs, were abundant [10], [26] but no wild hosts of tsetse were observed during the study.

### Collecting devices

Arrangements of electrocuting grids were used to assess the responses of tsetse to various visual baits [27]. Two types of electrocuting grid were used:-

1. Electric targets (henceforth termed E-targets) consisted of a panel of black cotton cloth sandwiched between two grids of fine copper wire (0.2 mm diameter, 8 mm apart and stained black by application of black ink); the grids electrocuted tsetse as they landed on the cloth.
2. Electric nets (henceforth E-nets) comprised a panel of fine black polyester net (Quality no. 166, Swisstulle, Nottingham, UK), also sandwiched between two grids of wires. The fine polyester net of the E-net and the electrocuting grids are effectively invisible to tsetse [27], [28], so that tsetse collided with the E-net and hence were captured.

**Figure 1:**
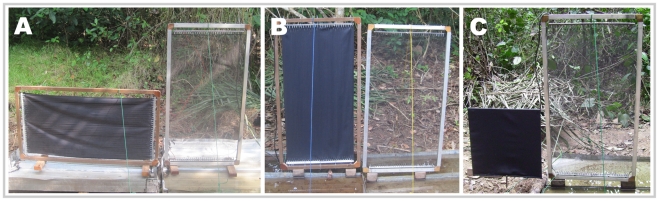
Examples of artificial baits and collecting devices. (A) 0.5×1.0 m horizontal E-target accompanied by a 0.5×1.0 m E-net, used in experiments A and B. (B) 0.5×1.0 m vertical E-target accompanied by a 0.5×1.0 m E-net, used in experiments A and B. (C) 0.5×0.5 m “inert” target accompanied by a 0.5×1.0 m E-net, used in experiments D, E and F.

The E-target and E-nets were often operated side-by-side and thus tsetse that approached the E-target but did not land on it were often caught by the adjacent E-net. The grids were mounted on metal trays (5 cm deep) containing soapy water, which caught and retained electrocuted flies. E-targets were of varying dimensions, but E-nets were always 0.5 m wide ×1.0 m high (see Fig. 1 for examples of arrangements of electrocuting grids).

### Visual targets

Studies of the numbers of tsetse attracted to and landing on small (e.g., 0.1×0.1 m) E-targets face the problem that the framework which supports the grid of wires may itself be a source of visual stimuli. To overcome this, we conducted a second series of experiments where we placed an E-net next to various panels of black cotton cloth mounted on a simple wire frame. These panels were not enclosed in a grid, and hence, tsetse that landed on it were not caught. Instead, the catch from the flanking E-net provided a relative measure of the numbers of tsetse attracted to the target (Fig. 1C). The visual targets are referred to as ‘inert targets’ to distinguish them from the electrified E-targets.

### Experimental design and analyses

All field experiments were carried out for a 4 h period between 09:30 hours and 14:30 hours local time, when *G. palpalis* and *G. fuscipes* are most active [29], [30]. Visual baits were compared over 10–21 days in a series of Latin-squares, of days×sites×treatments. Experimental sites was at least 100 m apart. To facilitate comparisons across species and experiments, all experiments included a standard treatment comprising an E-target (1 m×1 m) flanked by an E-net (1 m×1 m).

#### Vertical vs. horizontal oblongs

In Côte d'Ivoire, the responses of tsetse to vertical and horizontal oblongs was assessed by comparing the catches from E-targets that were: (1.) 0.5×1.0 m (Fig. 1A and 1B), (2.) 0.25×0.50 m or (3.) 0.125×0.25 m (Experiment A) arranged with their long axis arranged vertically or horizontally and the base on the ground. All E-targets were accompanied by an E-net. A similar experiment was conducted in the DRC, where we compared the catches from oblong (0.5×1.0 m or 0.125×0.5 m) E-targets with (Experiment B) or without (Experiment C) accompanying E-nets.

#### Vertical oblong vs. squares

In Côte d'Ivoire, we compared the numbers of tsetse attracted to four black inert targets of various size and shape: (1.) 0.35×0.71 m high, (2.) 0.5×0.5 m (Fig. 1C), (3.) 0.5 m×1.0 m, and (4.) 0.71×71 m (Experiment D).

#### Size

The effect of target size was studied in Côte d'Ivoire (Experiment E) and DRC (Experiment F) by comparing the numbers of tsetse attracted to square targets of decreasing size: (i) 1.0×1.0 m, (ii) 0.75×0.75 m, (iii) 0.5×0.5 m (Fig. 1C), (iv) 0.25×0.25 m, (v) 0.1×0.1 m, and (vi) no target. An E-net was placed adjacent to each target to assess the numbers of tsetse attracted but the targets themselves were not electrified.

### Statistical analyses

#### Catches

The daily catches were normalized and variances homogenized using a log10(*n*+1) transformation, and then subjected to analysis of variance using GenStat 11 edition (version 11.1.0.1504). For each experiment, the shape (i.e., vertical oblong, horizontal oblong, square) and size (i.e., surface area) of a target were specified as factors, and analyses of variance were carried out to assess whether these factors, and the interaction between them, had a significant effect on catch. Detransformed means are reported accompanied by their respective transformed mean and standard error of the difference (SED) between means.

#### Landing responses

To assess whether target size and/or shape influenced landing response, the proportion of tsetse that landed on an E-target was quantified by expressing the catch from an E-target as a proportion of the total (E-target+E-net). These data were analysed by logistic regression, with the catch from the E-target being specified as the *y*-variable and the total catch (E-target+E-net) as the binomial denominator. Days, sites and treatments (e.g. shape, size) were specified as factors. The statistical significance of differences in the proportion of tsetse landing on the target or entering a trap was assessed by removing the treatments factor from the full model. The significance of changes in deviance was assessed by χ2 or, if the data were overdispersed, an *F*-test following re-scaling [31]. The SE is asymmetric about the mean, and thus mean percentages are accompanied by the larger back-transformed SE. For all analyses, the level of significance was established at *P*<0.05.

#### Catch density

The practical aim of the study was to provide a rational basis for designing cost-effective targets. For this purpose, it is useful to consider the numbers of tsetse killed per unit area of the target, henceforth termed the ‘catch density’. The catch density for each target was calculated by dividing the mean daily catch (*x*) by the area (m2) of the target (E-target or inert target). Note that we do not include the area of the E-net in this calculation. For example, if E-nets (0.5 m^2^) placed next to ‘inert’ targets of, say, 0.1 m^2^ and 1 m^2^ caught respectively 20 and 100 tsetse/day, then the catch densities would be 20/0.1 = 200 tsetse/m2 and 100/1 = 100 tsetse/m2, respectively. To allow comparisons across experiments, catch densities were expressed as a proportion of the mean daily catch of the Standard target and this value is termed the Catch Density Index. Hence, if in the above example, a Standard target caught 200 tsetse/day, then the above Catch Density Indices would be 200/200 = 1 and 100/200 = 0.5, respectively. Indices greater or less than unity imply that the catch density is more or less than the standard.

## Results

There were no clear or consistent differences in the responses of male and female tsetse and so the results for the pooled (males+females) catches are presented.

### G. p. palpalis

#### Vertical vs. horizontal

**Figure 2:**
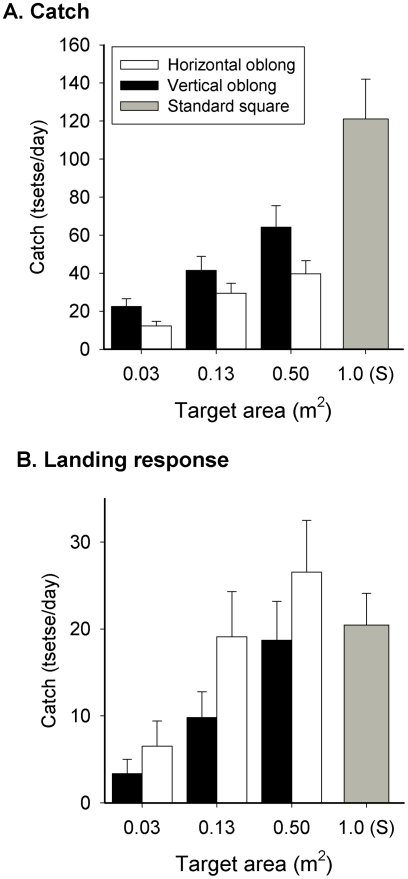
Response of *G. p. palpalis* to targets of different shape. (A) Detransformed mean catch (+SED) and (B.) landing response (+SE) of *G. p. palpalis* from vertical (solid bars) or horizontal oblongs (open bars) or the standard square target (S, grey bar). Oblongs were 0.125×0.25 m (surface area = 0.03 m^2^), 0.25×0.50 cm (0.13 m^2^), or 1×0.5 m (0.5 m^2^). All oblong targets were adjacent to an E-net, 0.5 m wide×1.0 m high. The Standard comprised a 1×1 m black E-target accompanied by a 1×1 m E-net.

Vertical-oblong targets caught consistently more (1.4–1.8×) tsetse than horizontal ones of the same surface area (Fig. 2A). The data were subjected to analysis of variance with shape and size specified as factors. Both factors had a highly significant effect on catch (Shape: *F*_1,61_ = 23.6, *P*<0.001; Size: *F*_2,61_ = 45.1, *P*<0.001) but there was no significant interaction between them (*F*_2,59_ = 0.5, *n.s*). All oblongs caught significantly fewer tsetse than the standard target with the largest vertical oblong (area = 0.5 m^2^) catching about half (64 tsetse/day) that of the standard square target (121 tsetse/day).

The percentage of tsetse caught on the target also increased with target size but, for each size, the landing response was greater on the horizontal-oblong (Fig. 2B). As with the catch data, shape (*F*_1,61_ = 18.7, *P*<0.001) and size (*F*_2,61_ = 32.7, *P*<0.001) had a highly significant effect on the landing response but there was no interaction between them (*F*_2,59_ = 0.9, *n.s*).

#### Vertical vs. square

**Figure 3:**
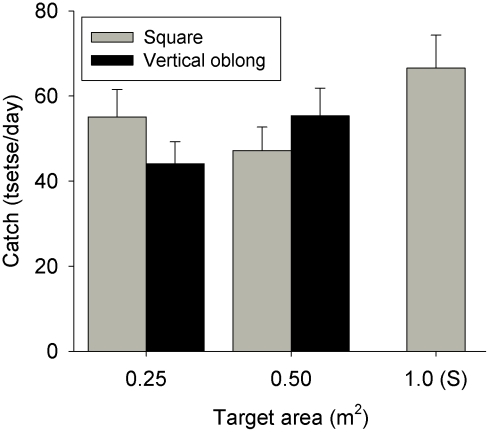
Detransformed mean catches (+SED) of *G. p. palpalis* attracted to the vicinity of vertical oblong (solid bars) or square (grey bars). Oblongs were 0.71×0.35 m (surface area = 0.25 m^2^) or 1×0.5 m (0.5 m^2^) and the matching square targets had dimensions of 0.5×0.5 m or 0.71×0.71 m, respectively.

The Standard square target caught more *G. p. palpalis* and elicited a stronger landing response than the oblongs, but this may be because it had a larger surface area rather than its shape *per se*. To test this hypothesis, we compared the catches from vertical oblongs and squares of equivalent surface area. The results (Fig. 3) show that there was no significant difference in the numbers attracted to squares and vertical oblongs of equal surface area (*F*_1,39_ = 0.2, *n.s.*). Thus square and vertical oblong shapes are equally attractive. The standard (1×1 m) target caught 67 tsetse/day compared to 47 tsetse/day for the 0.5 m^2^ square target (i.e. 0.71×0.71 m) and 55 tsetse/day for the 0.25 m^2^ one (i.e., 0.5×0.5 m). Thus while smaller targets caught fewer tsetse, the reduction was relatively slight (∼25%).

#### Effect of size

**Figure 4:**
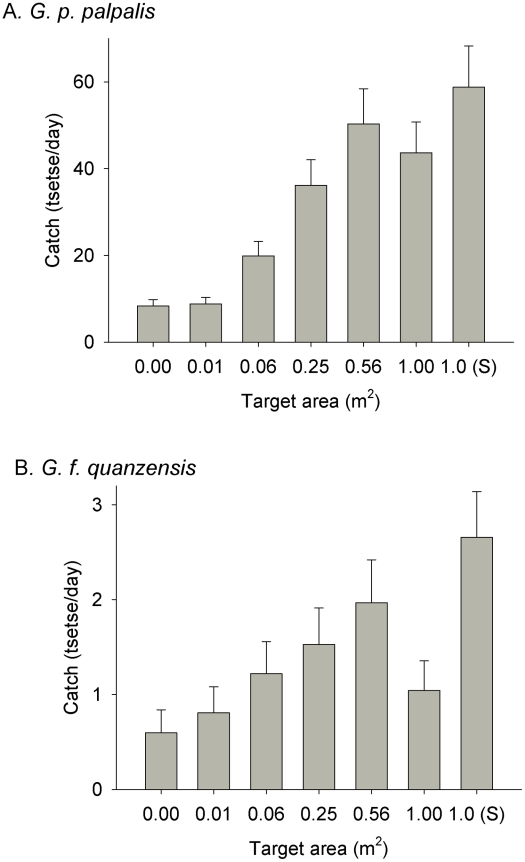
Attraction of tsetse to different sized targets. Detransformed mean catches (+SED) of (A) *G. p. palpalis* and (B) *G. f. quanzensis* attracted to square targets of various size. All targets were accompanied by an E-net 0.5 m wide×1 m high. S is the Standard, comprising an E-target (1×1 m) accompanied by an E-net (1×1 m).

The effect of size was examined further by comparing the numbers of tsetse attracted to the vicinity of square targets of various size ranging between 0.01 m^2^ (0.1×0.1 m) to 1.0 m^2^ (1×1 m). The results (Fig.4A) show that there was no significant difference between no target (i.e., an E-net without any adjacent target) and the smallest target (0.01 m^2^). Thereafter, catch increased with size but plateaued for targets with a surface area between 0.5 m^2^ and 1 m^2^.

### G. f. quanzensis

#### Vertical vs. horizontal

**Figure 5:**
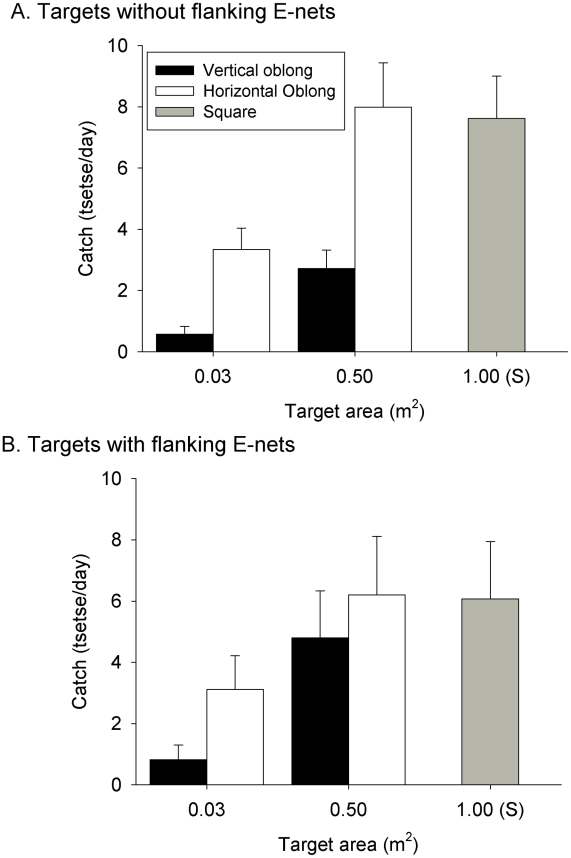
Attraction of tsetse to different shaped targets. Detransformed mean catch of *G. f. quanzensis* (+SED) from vertical (solid bars) or oblong (open bars) E-targets operated (A.) alone or (B.) with flanking E-nets. Oblongs were 0.125×0.25 m (surface area = 0.03 m^2^) or 1×0.5 m (0.5 m^2^) and accompanying E-nets were 0.5 m wide×1.0 m high. Both experiments included a Standard target (grey bar) consisted of a square (1×1 m) E-target accompanied by a 1×1 m E-net.

In contrast to the results for *G. p. palpalis,* horizontal oblongs were consistently more attractive than vertical ones for *G. f. quanzensis* (Fig. 5A). For E-targets not accompanied by an E-net, shape (*F*_1,24_ = 77.5, *P*<0.001) and size (*F*_1,24_ = 54.4, *P*<0.001) had highly significant effects on catch but there was no interaction between these factors (*F*_1,23_ = 0.4, *n.s.*). Similarly, for the E-targets accompanied by a flanking E-net (Fig. 5B), shape (*F*_1,24_ = 7.8, *P*<0.01) and size (*F*_1,24_ = 21.6, *P*<0.001) were highly significant but there was no interaction between them (*F*_1,23_ = 2.8, *n.s.*). Overall, the horizontal oblongs without or with accompanying E-nets caught ∼1.7–3.4× more *G. f. quanzensis* than vertical oblongs, and the bigger (0.5 m^2^) targets caught twice as many tsetse as small (0.03 m^2^) ones.

#### Effect of size

As with *G. p. palpalis*, the effect of size was examined by comparing the numbers of tsetse attracted to square targets ranging in size between 0.01 m^2^ (0.1×0.1 m) to 1.0 m^2^ (1×1 m). The results (Fig.4B) show that effect of size for *G. f. quanzensis* is very similar to that for *G. p. palpalis,* despite the large difference in the absolute size of catches which is merely a reflection of the total number of flies at each site (∼0.5–3 *G. f. quanzensis*/day vs. 8–59 *G. p. palpalis*/day): the catch increased with size up to ∼0.5 m^2^ where it plateaus. For both *G. p. palpalis* and *G. f. quanzensis*, the 1 m^2^ target caught less than the standard, which also had a 1 m^2^ E-target. This may be because the standard target had a larger flanking E-net (1 m^2^ vs. 0.5 m^2^).

### Catch density

**Figure 6:**
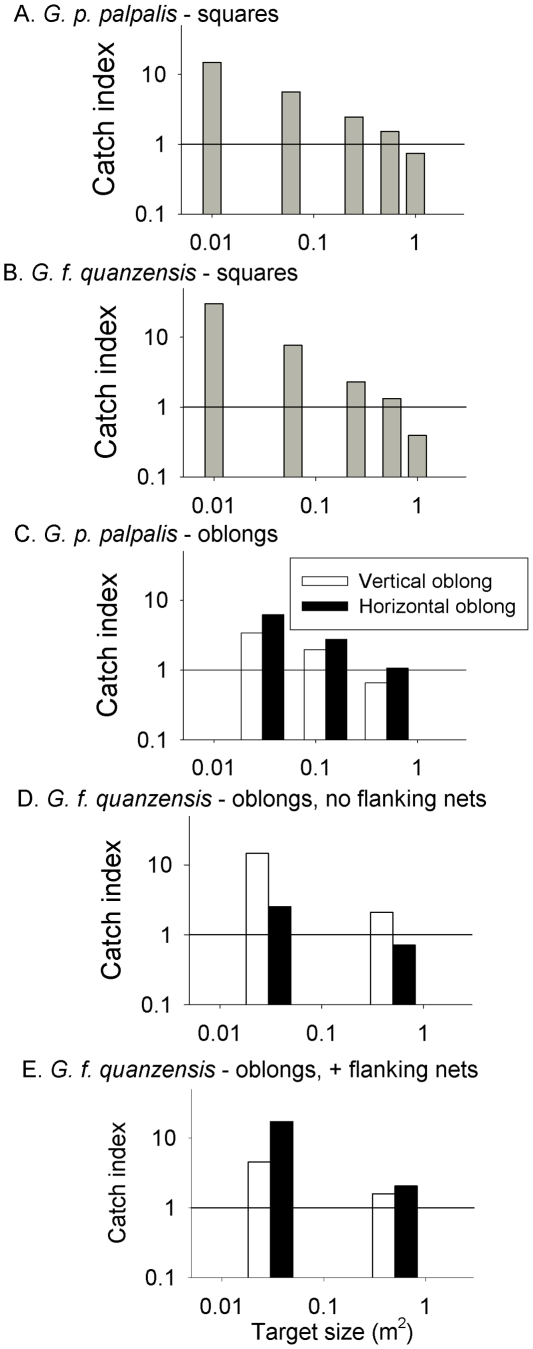
Proportional catch of tsetse on different shaped targets. Mean catch density (tsetse/m^2^) of square or oblong targets expressed as a proportion of that from a standard target for (A) *G. p. palpalis or* (B) *G. f. quanzensis* attracted to squares, and (C) *G. p. palpalis* or (D,E) *G. f. quanzensis* attracted to oblongs. All targets were accompanied by flanking e-nets except D. The horizontal line in each figure denotes the catch index of the Standard.

For both species, larger targets caught more tsetse but the increase was relatively slight. For instance, increasing from a 0.06 m^2^ to a 1 m^2^ target only doubled the catch of *G. p. palpalis* and had an even smaller effect for *G. f. quanzensis*. The results (Fig. 6) show that for all targets, irrespective of shape and/or species, the catch density index decreases as the size of the target increases showing that it is more cost effective for control programmes to produce large numbers of small targets from the material available.

## Discussion

The present results show that for the Palpalis-group species *G. p. palpalis* and *G. f. quanzensis*, the numbers of tsetse attracted to a bait is influenced by the bait's size and shape. For both species very small objects (∼0.01 m^2^ in surface area) do not appear to be detected by tsetse. As the object increases from 0.06 m^2^–0.56 m^2^, the catch doubles but further increases up to 1 m^2^ in size do not appear to increase the catch significantly. In contrast to size responses to shape differ between the species: *G. f. quanzensis* is attracted more to horizontal oblongs than vertical ones, whereas *G. p. palpalis* is more attracted to vertical oblongs.

### Practical implications

We demonstrate that catch increases with target size but the increase is not in proportion to the increase in target surface area. Hence, paradoxically, the numbers of tsetse killed per area of cloth, and by implication tsetse killed per dollar, decreases with increasing target size. The response to size shown here is similar to that of other Palpalis group species [12]–[14]. In particular, there is only a relatively modest doubling in the number of tsetse attracted to large (1 m^2^) targets versus small (e.g 0.25×0.25 m) ones. Given that tiny targets plus flanking nets (0.25×0.5 m) use 1/8^th^ and 1/24^th^ the amount of materials required respectively for the large 1 m^2^ targets or biconical traps, which are currently used in control programmes, it is clear that considerable savings in costs are gained by using tiny targets in control operations. It is interesting to note that as the size of a target is increased, the number of tsetse attracted per unit area of target decreases for Palpalis-group species but increases for Morsitans-group tsetse [15]. We are only just beginning to understand the fundamental differences in host location behaviour between the groups.

Beyond this general principle, the present results should be used with caution in identifying the optimal size of target. Taking the present results at face value for instance, a very small target (0.01 m^2^) had the highest catch density index, and since an E-net without any target caught some tsetse it has an infinitely high catch density. Concluding that no target will be most cost-effective is clearly nonsense! It is likely that since Palpalis-group tsetse are very sensitive to small targets, the structures associated with electric grids (transformer, 12 V battery, supporting frame of the grid) attract some tsetse, despite our efforts to make these items as inconspicuous as possible. The 0.01 m^2^ target did not catch significantly more tsetse than no target and hence it seems that tsetse are not responding to targets of 0.1×0.1 m or smaller. The 0.25×0.25 m target did catch significantly more tsetse than no target and this probably represents the smallest target that might be considered. The catch density declines steadily as size increases and there is no evidence that more tsetse were attracted to a 1 m^2^ target than a 0.5 m^2^ one. Hence a target in the region of 0.25×0.25 to 0.5×0.5 m seems likely to be optimal. The performance of these small targets is crucially dependent on the presence of a flanking net: while Palpalis-group tsetse are attracted to small objects, few land on them and hence a flanking net treated with a insecticide is essential for killing flies that visit but do not land. Recent results [12]–[14] suggest that a flanking net equal in size to the target is optimal.

The present results suggest that while there are marked differences in the responses of *G. f. quanzensis* and *G. p. palpalis* to oblongs, squares were as attractive as oblongs providing each had an equivalent surface area. Hence, square targets are likely to be effective to a wider range of species rather than, say, having vertical oblong targets for *G. p. palpalis* and horizontal ones for *G. f. quanzensis*.

### Host-seeking behaviour

The present results along with those of Rayaisse *et al.* [14] are the first demonstration of a tsetse species (*G. p. palpalis*) being attracted to a vertical oblong in preference to a horizontal one. For all other species, vertical and horizontal oblongs are either equally attractive (*G. m. morsitans* and *G. pallidipes*, [12]; *G. f. fuscipe*s, [21]) or horizontal oblongs are more attractive (*G. m. morsitans* and *G. pallidipe*s, [32]; *G. f. quanzensis*, present study). Previously, the preference for horizontal oblongs has been assumed to be related to the general shape of the mammalian hosts of tsetse [33]. It is therefore remarkable that just one species should not display this response. It is tempting to speculate that this is related to its anthropophilic feeding habit [22]; responding to an upright form may be adaptive for day-active Diptera that feed on humans.

The present study found that while *G. p. palpalis* was attracted to vertical oblongs, horizontal oblongs elicited a stronger landing response. Studies of the responses of Morsitans-group tsetse have also found marked differences in the orientation and landing responses of tsetse to shape: for *G. m. morsitans* and *G. pallidipes*, horizontal and vertical oblongs are equally attractive but the former elicits a stronger landing response. For *G. f. quanzensis* too, the horizontal oblong E-targets caught 7x more tsetse than the vertical ones when they were not accompanied by flanking E-nets, compared to a two-fold difference when the E-nets were present. This suggests that the horizontal targets are more attractive and elicit a stronger landing response. There are clearly many subtle inter-specific differences in the responses of tsetse to target shape.
